# Draft Genome Sequence of Escherichia albertii Strain Mex-12/320a, Isolated from an Infant with Diarrhea and Harboring Virulence Genes Associated with Diarrheagenic Strains of Enteropathogenic Escherichia coli

**DOI:** 10.1128/MRA.00208-19

**Published:** 2019-07-03

**Authors:** Samantha Maldonado-Puga, Mario Meza-Segura, Adriana Becerra, Mussaret B. Zaidi, Teresa Estrada-Garcia

**Affiliations:** a Department of Molecular Biomedicine, CINVESTAV-IPN, Mexico City, Mexico; b Infectious Diseases Research Unit, Hospital General O’Horan, Merida, Mexico; c Department of Epidemiology and Biostatistics, Michigan State University, Lansing, Michigan, USA; University of Maryland School of Medicine

## Abstract

Escherichia albertii is an emerging human enteropathogen. We report the draft genome sequence of E. albertii strain Mex-12/320a, isolated from an infant with diarrhea. The presence of the pathogenic island O122/IE6 and the *nleA* gene, previously found in diarrheagenic enteropathogenic Escherichia coli strains, suggests that *E. albertii* may cause acute diarrhea.

## ANNOUNCEMENT

Escherichia albertii is an emerging human enteropathogen which belongs to the attaching and effacing (A/E) family of gastrointestinal pathogens that include well-known human pathogens such as enteropathogenic Escherichia coli (EPEC) and enterohemorrhagic E. coli (1). The genes responsible for the A/E phenotype are carried on a pathogenicity island (PAI) known as the locus of enterocyte effacement (LEE), which encodes several effector proteins that are all injected into the host cell by the type III secretion system (T3SS) (2). Other non-LEE-encoded (NLE) EPEC effectors are translocated by the T3SS (1). EPEC non-LEE genes, such as *nleA* and those harbored in the PAI O122/IE6 (*efa1* or *lifA*, *espL*, *nleB*, and *nleE*), are significantly more prevalent among EPEC strains isolated from children with diarrhea than in those without (3–5). Most of these genes are involved in bacterial evasion of the host innate immune response (6, 7).

We report the draft genome sequence of E. albertii strain Mex-12/320a, a lactose-negative E. coli strain that was isolated from the stool of a 2-year-old girl with acute diarrhea. She was admitted to a hospital in Yucatan, Mexico, in 2012 for dehydration after 4 days of illness with 4 stool movements per day and 12 episodes of vomiting. This strain was isolated and characterized as previously described (8), was originally classified as atypical EPEC, and was the sole pathogenic isolate identified in the fecal sample. It was subsequently reclassified as *E. albertii* by a multiplex *in silico* PCR that discriminates between different Escherichia species using primers for the DNA-binding transcriptional activator of a cysteine biosynthesis gene (393-bp product) that appears to be specific to this species (9).

One colony from MacConkey agar was selected and incubated in LB broth for 18 h at 37°C without agitation. Genomic DNA was extracted using a phenol-chloroform protocol, prepared as Illumina sequencing libraries using the Nextera XT kit, and sequenced by the GAIIx system (Illumina, San Diego, CA, USA), generating 3,148,027 reads. Quality control, trimming, and filtering of raw sequencing data were performed with Trim Galore v0.4.1 (https://www.bioinformatics.babraham.ac.uk/projects/trim_galore/). The genome was assembled *de novo* using the SPAdes genome assembler v3.9.1 (http://cab.spbu.ru/software/spades/), and the accuracy of the genome assemblies was evaluated with REAPR 1.0.16 (10). Protein-coding sequences and RNA genes were predicted, and functional annotation was performed with Prokka v1.11. Preassembled contigs were scaffolded using SSPACE_Standard_v3.0.pl (11), and gaps were closed with GapFiller_v1-10 (12). Default parameters were used for all software during the assembly and annotation process.

The draft chromosome sequence of *E. albertii* strain Mex-12/320a consists of 4,941,694 bp in 268 scaffolds, with an *N*_50_ value 130,167 bp, a maximum scaffold size of 341 kbp, and 49.54% G+C content. The genome contains 5,090 predicted genes, of which 4,763 are coding sequences (CDSs), 5 are rRNA (*rrn*) operons, and 79 are tRNA genes. The genome sequence confirmed the presence of not only the LEE genes but also *nleA* and *cdt-II* (cytolethal distending toxin II) virulence genes previously found in other *E. albertii* strains (13, 14). To the best of our knowledge, this is the first report of the presence of the complete PAI O122/IE6 (*efa1* or *lifA, espL, nleB,* and *nleE* genes) in an *E. albertii* strain.

This work confirms that the *E. albertii* Mex-12/320a genome harbors virulence genes that have been previously reported in EPEC isolates from children with diarrhea. This finding adds to the growing body of evidence that *E. albertii* is an emerging pathogen of diarrheal disease (15) and that *nleA* and the PAI O122/IE6 are virulence markers of A/E *Escherichia* pathogens. This report, in conjunction with those for other genomes of *E. albertii* (13, 16, 17), will contribute to a greater understanding of the phylogeny and evolution of *Escherichia* species and to the identification of new virulence factors among A/E bacteria.

### Data availability.

The draft genome assembly of *E. albertii* strain Mex-12/320a was deposited in DDBJ/ENA/GenBank under the accession number SIZV00000000 and BioProject number PRJNA523447. Sequencing reads for *E. albertii* strain Mex-12/320a have been deposited in the NCBI Sequence Read Archive (SRA) under the accession number SRR9024355.
